# Production of Biosurfactant by *Streptomyces luridus* So3.2 Using Commercial and Recycled Frying Oils in a Stirred-Tank Bioreactor

**DOI:** 10.3390/ijms27156672

**Published:** 2026-07-26

**Authors:** Claudio Lamilla, David Troncoso, Daniel Martínez-Cisterna, Edward Hermosilla, María Cristina Diez, Heidi Schalchli, Gabriela Briceño, Olga Rubilar

**Affiliations:** 1 Scientific and Technological Bioresource Nucleus (BIOREN), Universidad de La Frontera, Av. Francisco 7 Salazar 01145, Casilla 54-D, Temuco 4811230, Chile; 2 Centro de Investigación Biotecnológica Aplicada al Medio Ambiente (CIBAMA), Universidad de La Frontera, Av. Francisco Salazar 01145, Casilla 54-D, Temuco 4811230, Chile; daniel.martinez@ufrontera.cl (D.M.-C.); cristina.diez@ufrontera.cl (M.C.D.); heidi.schalchli@ufrontera.cl (H.S.); gabriela.briceno@ufrontera.cl (G.B.); 3 Programa de Doctorado en Ciencias de Recursos Naturales, Universidad de La Frontera, Av. Francisco Salazar 01145, Casilla 54-D, Temuco 4811230, Chile; d.troncoso03@ufromail.cl; 4 Instituto de Ciencias Aplicadas, Facultad de Ingeniería, Universidad Autónoma de Chile, Temuco 4810101, Chile; edward.hermosilla@uautonoma.cl; 5 Departamento de Ciencias Químicas y Recursos Naturales, Facultad de Ingeniería y Ciencias, Universidad de La Frontera, Av. Francisco Salazar 01145, Temuco 4780000, Chile; 6 Departamento de Ingeniería Química, Facultad de Ingeniería y Ciencias, Universidad de La Frontera, Av. Francisco Salazar 01145, Temuco 4780000, Chile

**Keywords:** *Streptomyces luridus*, recycled frying oil, Antarctic bacterium, stirred-tank bioreactor

## Abstract

Biosurfactants are sustainable alternatives to petroleum-derived surfactants, yet their industrial application is often constrained by production costs and process efficiency. This study aimed to evaluate biosurfactant production by the psychrotolerant Antarctic bacterium *Streptomyces luridus* So3.2 using low-cost recycled frying oils under mild cultivation conditions and to validate the process in stirred-tank bioreactors. Cultivation conditions were optimized using response surface methodology (RSM), and the same optimized pH and carbon source concentration were subsequently applied in stirred-tank reactors at laboratory scales of 2 L and 20 L, while aeration (0, 0.5 and 0.9 vvm) and agitation speed (100, 150, and 200 rpm) were evaluated as reactor-operational variables. Filtered and centrifuged recycled frying oil yielded the highest biosurfactant performance (emulsification indices exceeding 80%, enhanced oil displacement, and surface tension values below 40 mN m^−1^) compared to commercial oils. Biosurfactant production was growth-associated, with detectable surface activity within the first 24 h. RSM identified optimal cultivation parameters at pH 8.0, 3% (*w*/*v*) inoculum, and 2% (*w*/*v*) oil concentration. The biosurfactant exhibited a critical micelle concentration (CMC) of 29 mg L^−1^ and a critical micelle dilution (CMD) of 43.2, yielding an estimated broth concentration of 1.25 g L^−1^. At the 2 L reactor scale, moderate aeration (0.5 vvm) combined with intermediate agitation (150 rpm) preserved high surface activity, yielding emulsification indices above 83%, oil displacement halos of 12.5 cm, and surface tension values as low as 35.5 mN m^−1^. This performance was also maintained during validation at 20 L. FTIR and TLC analyses indicated lipid- and peptide-associated functional groups. These findings were further complemented by HPLC, MALDI-TOF MS and genome-mining analyses (antiSMASH), revealing a complex molecular profile and multiple NRPS/NRPS-like biosynthetic gene clusters, supporting the interpretation of the recovered product as a putative lipopeptide-associated surface-active extract. Overall, this work demonstrates the feasibility of producing a biosurfactant-associated surface-active extract from recycled frying oils using *S. luridus* So3.2 under mild conditions.

## 1. Introduction

Biosurfactants are surface-active compounds produced by microorganisms that reduce surface and interfacial tension through their amphiphilic molecular structures [1]. Due to their biodegradability, low toxicity, and functional stability, biosurfactants have emerged as promising alternatives to synthetic surfactants, which are predominantly derived from petroleum sources and are often associated with environmental persistence and ecotoxicity [2]. These compounds are microbially produced amphiphiles that can be recovered by extraction, precipitation, or other downstream separation methods, without the need for chemical synthesis at any stage, thus being classified as naturally derived surfactants. While many biosurfactants are neutral or anionic, those containing amine functional groups may exhibit cationic properties [3]. As a result, biosurfactants have attracted increasing interest for applications across diverse industrial sectors, including environmental remediation, agriculture, food processing, pharmaceuticals, and personal care formulations [4,5].

In this context, the utilization of agro-industrial residues as carbon sources represents a key strategy to improve the economic feasibility and sustainability of biosurfactant production [6]. Recycling waste or used vegetable oils into biosurfactants offers an environmentally friendly alternative to synthetic surfactants while simultaneously addressing issues related to improper oil disposal and the high cost of refined substrates [7,8]. Recent studies have highlighted the technical feasibility, environmental benefits, and broad industrial applicability of biosurfactants derived from recycled frying oils [9]. Moreover, the valorization of lipid-rich waste contributes to the circular bioeconomy, considering that approximately 29 million tonnes of lipid waste are generated worldwide each year and can be repurposed as low-cost substrates for biosurfactant synthesis [10]. The use of recycled frying oils significantly reduces biosurfactant production costs while mitigating environmental impacts associated with improper oil disposal, reducing dependence on virgin oils and avoiding competition with food resources [11]. Various microorganisms, including *Pseudomonas*, *Bacillus*, *Serratia*, *Rhodococcus*, yeast, and *Streptomyces*, have been reported to convert recycled frying oils into biosurfactants such as rhamnolipids, lipopeptides, sophorolipids, and trehalolipids. Importantly, the type of oil and its fatty acid composition (e.g., rapeseed, soybean, canola, sunflower) strongly influence biosurfactant yield and properties [12,13]. However, these highly variable waste streams require identifying optimal pre-treatments such as filtration and centrifugation to remove inhibitory micro-particulates and ensure maximum substrate accessibility for microbial conversion.

In recent years, the biotechnological potential of Antarctic microorganisms has gained increasing recognition due to their ability to produce structurally diverse secondary metabolites under extreme environmental conditions. Several biosurfactant-producing Antarctic bacteria have been reported, including *Pseudomonas*, *Bacillus*, *Pantoea*, *Rhodococcus* and *Streptomyces* [14,15,16]. Among these genera, *Streptomyces*, a dominant group within the phylum Actinobacteria, is particularly well known for its abundance in soils and its capacity to synthesize a wide array of bioactive secondary metabolites, including biosurfactants. Biosurfactant production by *Streptomyces* species is influenced not only by the metabolic capabilities of the producing organism but also by the operational conditions of the bioprocess [17]. However, industrial implementation is often constrained by the need for controlled cultivation conditions, refined carbon sources, and elevated energy inputs [18]. These limitations have driven increasing interest in identifying robust microorganisms capable of efficient biosurfactant production under mild conditions using low-cost substrates, positioning *Streptomyces* as a promising microbial group for industrial and biotechnological applications [19,20].

*S. luridus* So3.2, isolated from Antarctic soil, has previously been reported as a producer of bioemulsifying compounds capable of emulsifying a wide range of hydrocarbons and oils, highlighting its potential for industrial applications. Its ability to utilize low-cost substrates and remain functional under harsh environmental conditions further supports its relevance for biotechnological exploitation [16,21]. Although this strain originates from an extreme environment, its ability to maintain metabolic productivity at 25 °C represents a practical advantage for bioprocess operation. However, evidence for this strain has remained limited to emulsification-based assays [16]. In the present study, this previous knowledge is extended by evaluating additional surface-active properties and reactor-scale performance, which together provide broader support for biosurfactant-associated activity.

Nevertheless, most available evidence for this strain remains limited to flask-based assays, which do not adequately reproduce the hydrodynamic and oxygen-transfer conditions that become critical under reactor operation [22]. In biosurfactant-producing systems, variables such as aeration, agitation, and working volume can strongly affect both metabolite synthesis and functional surface activity, making reactor-based validation necessary to determine whether the behavior observed at flask scale can be maintained under controlled conditions and increased working volumes [23,24]. Moreover, an integrated evaluation combining process optimization, physicochemical characterization, and complementary chemical evidence is required to more comprehensively assess the biosurfactant-producing potential of this Antarctic strain.

Given these considerations, the present study aimed to evaluate and validate a scalable biosurfactant production process using *S. luridus* So3.2 and low-cost recycled frying oils, identify the most suitable substrate processing methodology, assess production kinetics and evaluate cultivation variables through response surface methodology. Complementary chemical and molecular analyses were also conducted to obtain evidence regarding the nature of the surface-active extract. Finally, the optimized process was validated in stirred-tank bioreactors at working volumes of 2 L and 20 L, linking substrate selection and process evaluation with reactor-based production. Overall, this work contributes to the development of a more robust and sustainable bioprocess platform with potential applications across multiple industrial sectors.

## 2. Results

### 2.1. Carbon Source Screening for Biosurfactant Production

The effect of different vegetable oils on biosurfactant production by *S. luridus* So3.2 was evaluated using functional surface-activity assays, including emulsification index (E24%), oil displacement, and surface tension reduction.

**Figure 1:**
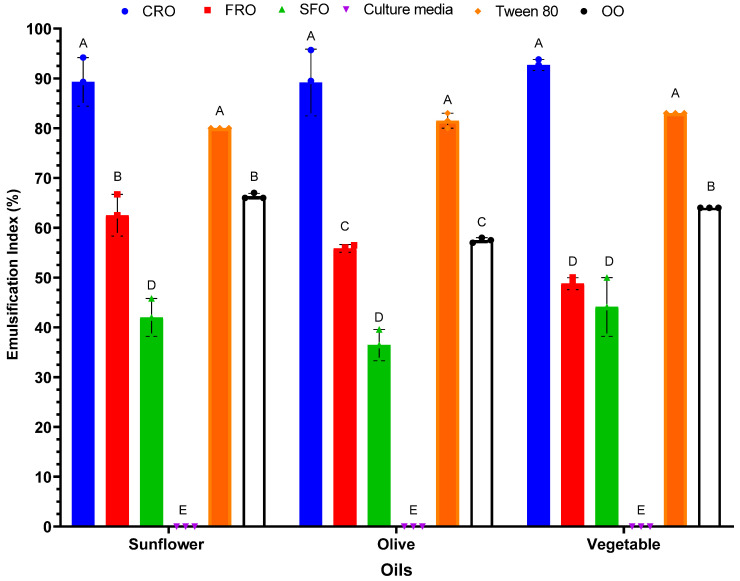
Determination of emulsification activity (E24%) in commercial sunflower, olive, and vegetable oils. The assay was performed using cell-free supernatants obtained from filtered and centrifuged recycled frying oil (CRO), olive oil (OO), filtered recycled frying oil (FRO), sunflower oil (SFO), and Tween 80 as the control. Different letters indicate significant differences (ANOVA, Tukey HSD test, *p* < 0.05).

Emulsification assays performed after 24 h revealed marked differences in E24% among the evaluated carbon sources (Figure 1). The highest emulsification activity was observed in cultures grown with filtered and centrifuged recycled frying oil (CRO), reaching values close to 90%, whereas filtered recycled frying oil (FRO) showed intermediate E24% values. In contrast, sunflower oil resulted in substantially lower emulsification. Tween 80 was included as a functional reference surfactant to benchmark the emulsification and surface-tension performance of the microbial extract. These results indicate that emulsification performance varied significantly depending on the oil source and was associated with microbial biosurfactant production rather than with the oil itself.

**Figure 2:**
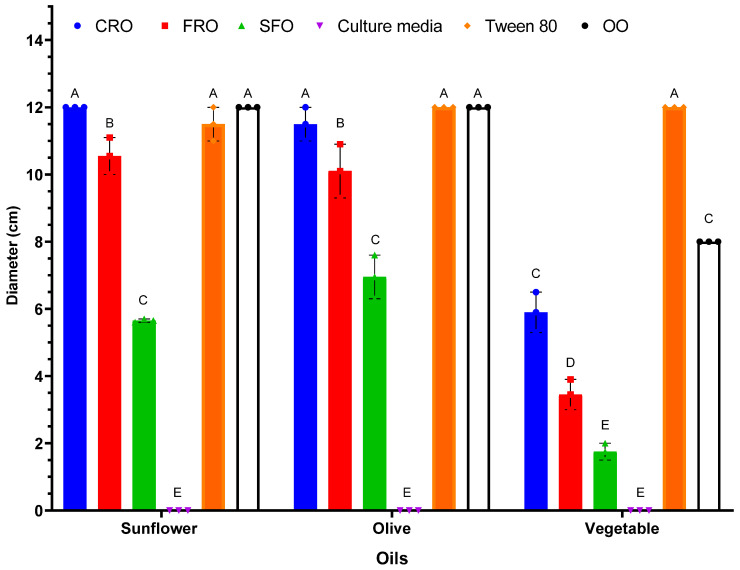
Determination of oil displacement in commercial sunflower, olive, and vegetable oils. The assay was performed using cell-free supernatants obtained from filtered and centrifuged recycled frying oil (CRO), olive oil (OO), filtered recycled frying oil (FRO), sunflower oil (SFO), and Tween 80 as the control. Different letters indicate significant differences (ANOVA, Tukey HSD test, *p* < 0.05).

Oil displacement assays further supported these findings (Figure 2). Cell-free supernatants obtained after 96 h of cultivation at 25 °C and 150 rpm from CRO cultures produced the largest clear zones at the oil–water interface, indicating enhanced interfacial activity. FRO cultures produced smaller displacement halos, whereas sunflower oil cultures showed limited oil displacement. No displacement was detected in the uninoculated medium control, confirming that the observed activity was exclusively linked to biosurfactant production by *S. luridus* So3.2.

Surface tension measurements were generally consistent with the emulsification and displacement results, although the lowest surface tension was observed in cultures grown with olive oil (33.5 ± 0.2 mN m^−1^), followed by FRO and CRO (37.6 ± 0.3 and 37.6 ± 0.2 mN m^−1^, respectively). The minimal medium control and distilled water exhibited surface tension values of 58.5 ± 0.0 and 70.1 ± 0.0 mN m^−1^, respectively. Tween 80 reduced surface tension to approximately 25 mN m^−1^.

Surface tension values and final pH measurements after 96 h of fermentation are summarized in Table 1. Recycled frying oil-based cultures (FRO and CRO) showed surface tension values below 38 mN m^−1^, confirming the presence of surface-active compounds in the cell-free supernatants. Final pH values ranged between 6.2 and 7.0 across all inoculated treatments, indicating that biosurfactant production occurred under near-neutral conditions.

Based on the combined results of emulsification index, oil displacement activity, and surface tension reduction, filtered and centrifuged recycled frying oil (CRO) was selected as the most suitable low-cost carbon source for subsequent kinetic studies and stirred-tank reactor experiments. This selection was based on its overall functional performance, particularly in emulsification and oil displacement.

### 2.2. Growth Kinetics and Biosurfactant-Associated Surface Activity

**Figure 3:**
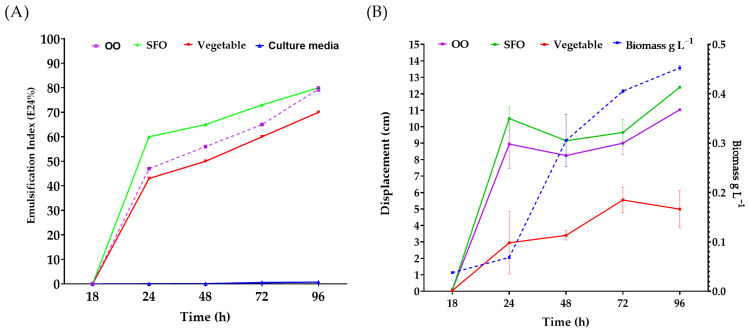
Growth kinetics and biosurfactant-associated surface activity of *S. luridus* So3.2 during shake-flask cultivation on selected vegetable oils. (**A**) Emulsification index (E24%) measured at 18 h, 24 h, and every 24 h thereafter until 96 h. (**B**) Oil displacement activity and biomass accumulation over the same cultivation period. Biomass is represented by the blue dotted line and corresponds to the right y-axis, while oil displacement values correspond to the left y-axis. OO, olive oil; SFO, sunflower oil; Vegetable, recycled frying oil-derived condition; culture medium, uninoculated control.

Emulsification and oil displacement activities increased progressively during cultivation (Figure 3A). Detectable emulsification was observed within the first 24 h of fermentation, indicating early biosurfactant production. Both E24% values and oil displacement diameters increased steadily over time and remained at high levels at later sampling points (80%), while those grown with commercial oils reached approximately 70%.

Growth kinetics of *S. luridus* So3.2 are shown in Figure 3B. A continuous increase in biomass was observed during the first 48 h, corresponding to the exponential growth phase. Cultures reached the stationary phase at approximately 72 h and maintained stable biomass levels thereafter. Media supplemented with recycled frying oil supported higher biomass accumulation compared to commercial oils.

The temporal profiles of growth and surface activity indicate that biosurfactant-associated activity developed concomitantly with active cell growth under the evaluated conditions.

### 2.3. CMC and CMD of the Biosurfactant

**Figure 4:**
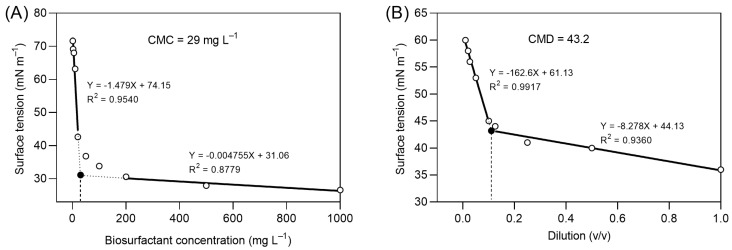
Determination of the critical micelle concentration (CMC) and critical micelle dilution (CMD) of the biosurfactant produced by *S. luridus* So3.2 grown on filtered and centrifuged recycled frying oil (CRO). (**A**) Surface tension as a function of biosurfactant concentration, used to estimate the CMC from the intersection of the two fitted linear regions. (**B**) Surface tension as a function of serial dilution of the cell-free supernatant, used to estimate the CMD from the breakpoint between two fitted linear regions. Dashed lines represent the linear regressions used for breakpoint estimation.

Surface tension decreased progressively with increasing biosurfactant concentration until reaching a plateau. The CMC and DMC values were estimated from the intersection of the two regression lines shown in Figure 4.

The critical micelle dilution (CMD) was estimated from the serial dilution profile of the cell-free supernatant (Figure 4B). The breakpoint between the two fitted linear regions corresponded to a CMD value of 43.2. Based on the relationship between CMC and CMD, the estimated biosurfactant concentration in the fermentation broth was calculated as approximately 1.25 g L^−1^.

These results indicate that the biosurfactant produced by *S. luridus* So3.2 exhibits high surface activity at relatively low concentrations.

### 2.4. Statistical Correlation of Surface Activity Parameters

**Figure 5:**
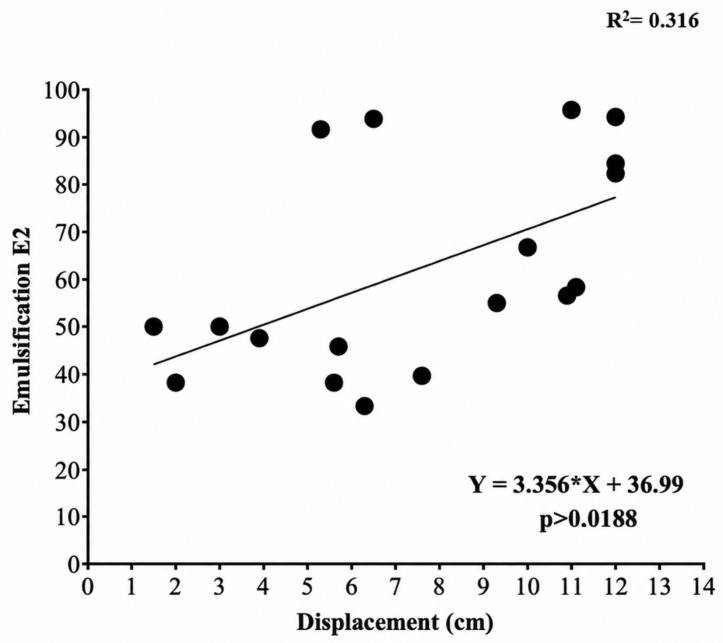
Linear regression analysis between emulsification index (E24%) and oil displacement activity in cell-free supernatants of *S. luridus* So3.2. Each point represents an individual experimental observation. The solid line indicates the fitted linear regression. The regression equation, coefficient of determination (R^2^), and significance level (*p* = 0.0188) are shown in the plot.

The relationship between emulsification activity (E24%) and oil displacement was evaluated to determine the degree of association between these two surface activity parameters. The statistical analysis is presented in Figure 5.

Linear regression analysis revealed a statistically significant positive correlation between the emulsification index (E24%) and oil-displacement diameters. The scatter plot showed an increasing trend, indicating that higher emulsification indices were associated with larger oil displacement zones. The regression model yielded a statistically significant *p*-value (*p* = 0.0188) and a coefficient of determination (R^2^ = 0.316), confirming that the observed correlation was not due to random variation.

These results demonstrate a consistent relationship between emulsification capacity and oil displacement activity across the evaluated conditions, supporting the use of both parameters as complementary indicators of biosurfactant-mediated surface activity.

### 2.5. RSM-Based Evaluation of Cultivation Variables

The Central Composite Design revealed marked variations in emulsification activity (E24%) as a function of pH, inoculum concentration, and recycled frying oil concentration (Table 2). Among the experimental runs, the highest emulsification response was obtained in Run 11 (pH 8.0, inoculum concentration 3% *w*/*v*, oil concentration 2% *w*/*v*), reaching an experimental E24 value of 82%, which was closely aligned with the value predicted by the model (81.3%). In contrast, the lowest emulsification index was observed in Run 10 (pH 7.0, inoculum concentration 1% *w*/*v*, oil concentration 1.5% *w*/*v*), yielding an experimental E24 of 52%, also in close agreement with the predicted value (53.0%).

The strong agreement between experimental and predicted E24% values across the experimental matrix indicates a high predictive capacity of the quadratic model. Replicated central-point experiments (Runs 14 and 15) produced similar emulsification indices, further supporting the reproducibility of the experimental design.

For the RSM analysis, E24% was used as the response variable for optimization, whereas oil displacement activity was evaluated as a complementary surface activity parameter. Analysis of variance (ANOVA) confirmed that the quadratic model was statistically significant (*p* = 0.0008), with a high coefficient of determination (R^2^ = 0.9828), indicating that more than 98% of the variability in emulsification activity was explained by the model. The lack-of-fit test was not significant (*p* = 0.6916), supporting the adequacy of the model to describe the experimental data (Table 3).

**Figure 6:**
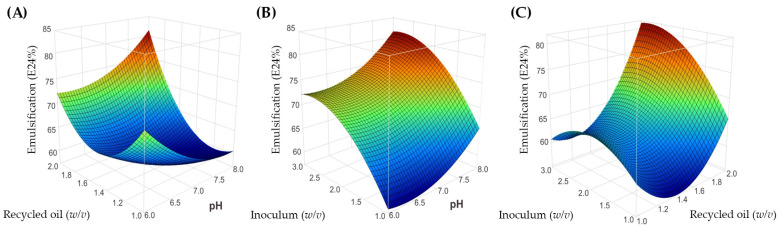
Three-dimensional response surface plots illustrating the interactive effects of cultivation variables on the emulsification index (E24%) of the biosurfactant produced by *S. luridus* So3.2. The graphs depict the cross-interactions between: (**a**) initial pH and recycled frying oil concentration (*w*/*v*); (**b**) initial pH and inoculum concentration (*w*/*v*); and (**c**) recycled frying oil concentration and inoculum concentration (*w*/*v*).

Figure 6 shows the three-dimensional response surface plots illustrating the interactions among pH, inoculum concentration, and recycled frying oil concentration on E24%. Evaluation of the regression coefficients indicated that inoculum concentration and recycled frying oil concentration exerted the strongest positive effects on emulsification performance, whereas pH had a comparatively lower influence within the evaluated range. The response surfaces showed that the highest E24% values were associated with higher inoculum and oil concentrations, particularly under slightly alkaline conditions.

The optimal cultivation conditions predicted by the RSM model were experimentally validated at the flask scale, resulting in emulsification indices consistently exceeding 80%, in agreement with the model predictions. The same optimized pH and carbon source concentration were subsequently applied in the 2 L and 20 L stirred-tank reactors to evaluate biosurfactant-associated surface activity under reactor conditions. These findings confirm the suitability of RSM as an effective tool for optimizing biosurfactant production by *S. luridus* So3.2 using recycled frying oil as a low-cost carbon source.

### 2.6. Reactor-Scale Biosurfactant Production

The production of biosurfactant by *S. luridus* So3.2 was first evaluated in a laboratory-scale stirred-tank reactor with a total capacity of 2 L and a working volume of 1 L to assess the effect of aeration and agitation on surface-active performance under controlled conditions. Selected combinations of aeration and agitation were evaluated as representative operating regimes after flask-level optimization, and the resulting surface activity parameters are summarized in Table 4.

Emulsification activity varied depending on the applied aeration and agitation regimes. The highest emulsification index (83.3%) was obtained at 0.5 vvm and 150 rpm, as well as at 0.9 vvm and 200 rpm. In contrast, the non-aerated regimes showed lower emulsification values, indicating that the selected operating conditions influenced the emulsification performance of *S. luridus* So3.2 at the reactor scale.

Oil displacement activity followed a similar trend. The maximum oil displacement halo (12.50 ± 0.86 cm) was observed at 0.5 vvm aeration and 150 rpm agitation, highlighting the relevance of this operating regime for surface-active performance. Lower displacement halos were observed under the other evaluated conditions. Surface tension measurements were consistent with these results, with the lowest value (35.5 mN m^−1^) also recorded at 0.5 vvm and 150 rpm, whereas higher values (up to 38.5 mN m^−1^) were obtained in the remaining treatments. Overall, these results indicate that the selected aeration/agitation regimes affected the surface-active behavior of *S. luridus* So3.2 in the stirred-tank reactor.

**Figure 7:**
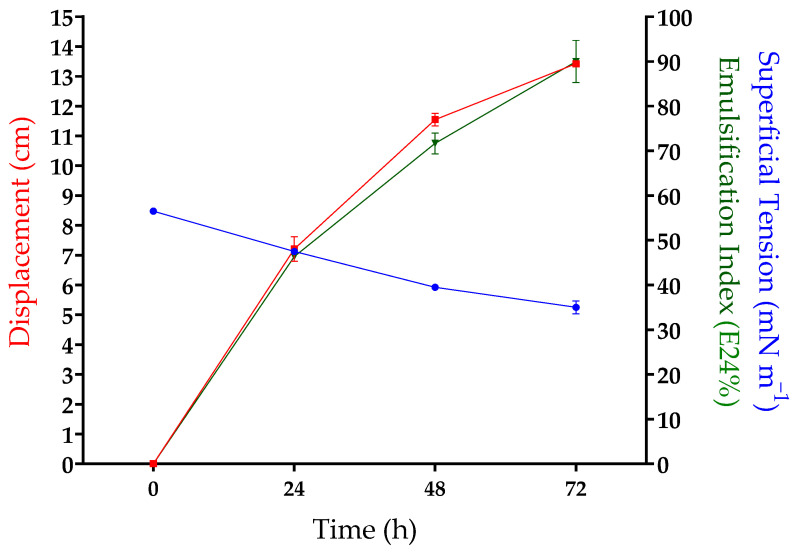
Temporal evolution of biosurfactant-associated surface activity during batch fermentation of *S. luridus* So3.2 in a 20 L stirred-tank reactor. Oil displacement activity (cm), emulsification index (E24%), and water surface tension (mN m^−1^) were monitored over 72 h of cultivation using cell-free supernatants.

Based on the optimized cultivation conditions identified at flask scale and maintained in the 2 L reactor, biosurfactant production was subsequently assessed in a 20 L stirred-tank reactor under batch conditions. The temporal evolution of surface activity parameters during the first 72 h of cultivation is shown in Figure 7. Emulsification activity increased progressively during fermentation, with detectable activity after 24 h and values exceeding 80% at 72 h. Oil displacement activity followed a similar temporal profile, while surface tension decreased throughout cultivation. These results indicate that the surface-active behavior observed at a smaller scale was maintained under the evaluated 20 L operating conditions.

Emulsification activity progressively increased during cultivation, with detectable activity observed after 24 h and values exceeding 80% at 72 h. Oil displacement activity exhibited a comparable temporal profile, with expanding clear zones as fermentation progressed, indicating increasing interfacial activity of the cell-free supernatants. In parallel, surface tension decreased steadily throughout the fermentation period, reaching minimum values comparable to those obtained in the laboratory-scale reactor.

The parallel trends observed in emulsification, oil displacement, and surface tension reduction indicate that the surface-active behavior observed in the 2 L reactor was maintained under the evaluated 20 L operating conditions. These results support the feasibility of biosurfactant-associated surface activity at increased working volume, although more detailed reactor-level characterization is still required.

**Figure 8:**
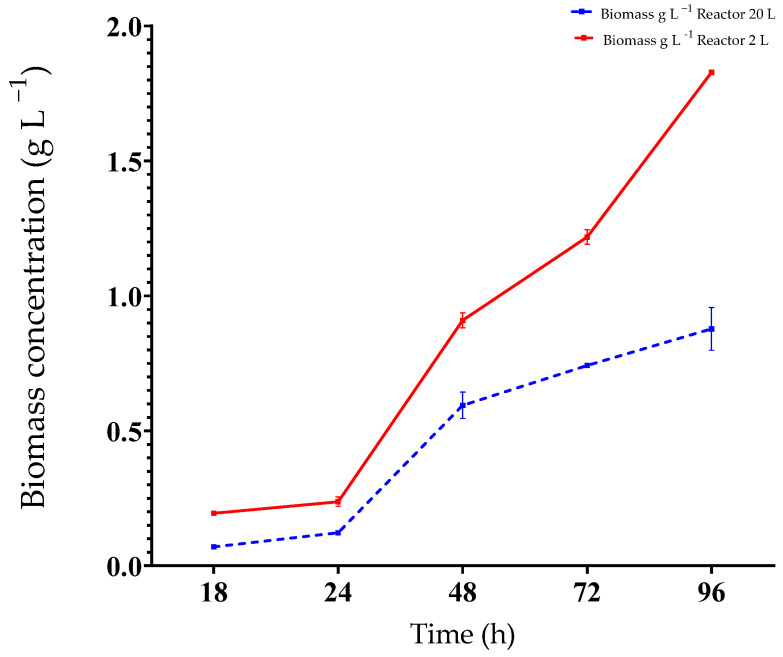
Biomass accumulation of *S. luridus* So3.2 during cultivation in 2 L and 20 L stirred-tank reactors. Biomass concentration (g L^−1^) was monitored at 18, 24, 48, 72, and 96 h. Data are presented as mean ± standard deviation.

Biomass accumulation was also compared between the 2 L and 20 L stirred-tank reactors throughout cultivation (Figure 8). In both systems, biomass increased progressively over time, confirming active microbial growth under the evaluated reactor conditions. However, the 2 L reactor consistently reached higher biomass concentrations than the 20 L reactor at all evaluated time points. At 96 h, biomass in the 2 L reactor reached approximately 1.83 g L^−1^, whereas the 20 L reactor reached approximately 0.88 g L^−1^. Despite this difference in biomass accumulation, both reactors maintained biosurfactant-associated surface activity, indicating that surface-active performance was preserved at increased working volume even under lower biomass production in the 20 L system.

### 2.7. Chemical, Analytical, and Genomic Characterization of the Surface-Active Extract

**Figure 9:**
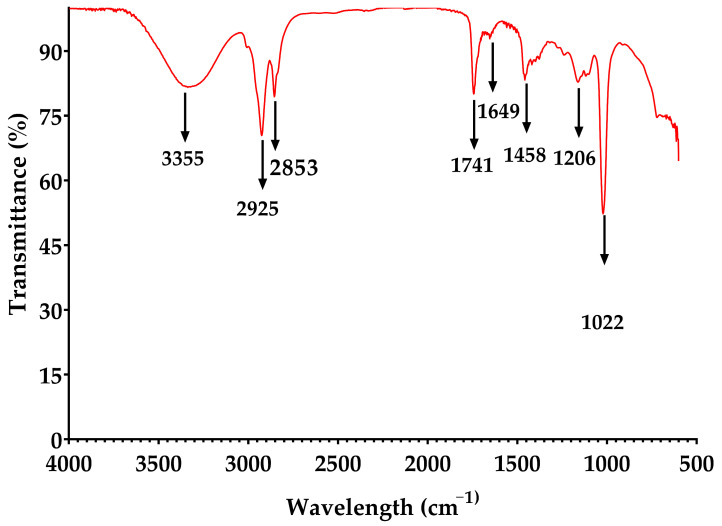
FTIR spectra of the biosurfactant obtained from strain So3.2 using recycled frying oil as the sole carbon source.

FTIR analysis revealed characteristic absorption bands associated with lipid- and peptide-associated functional groups (Figure 9). A broad band observed at approximately 3355 cm^−1^ corresponds to O-H and N-H stretching vibrations. Bands at 2925 and 2853 cm^−1^ were attributed to aliphatic C-H stretching, indicating the presence of long hydrocarbon chains. The absorption peak at 1741 cm^−1^ was assigned to ester or carboxylic C=O stretching, while the band at 1649 cm^−1^ corresponded to amide I vibrations, suggesting peptide linkages. Additional bands at 1458 cm^−1^ (CH_2_ bending) and in the 1206–1022 cm^−1^ region (C-O stretching) were also detected.

**Figure 10:**
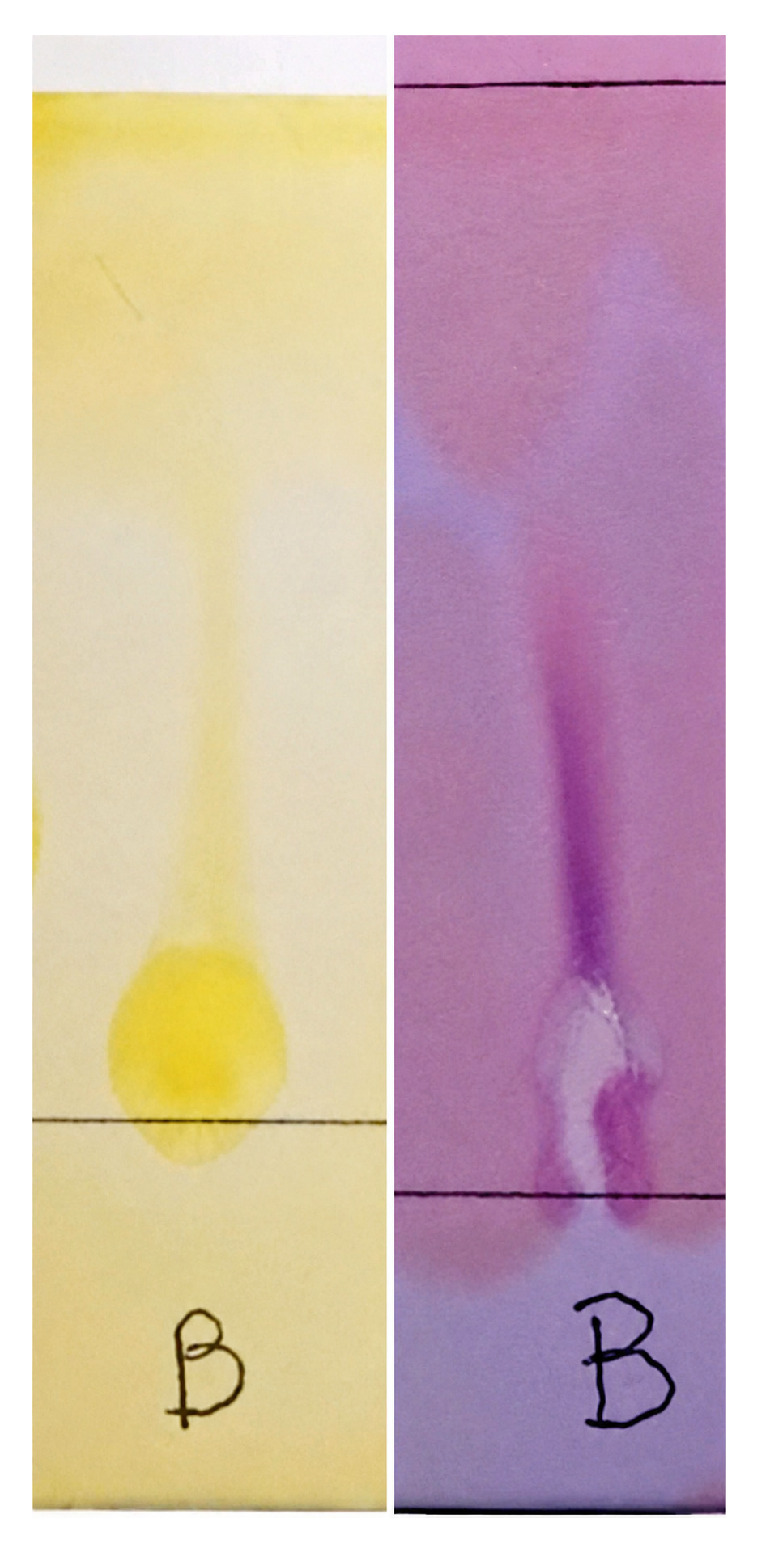
Thin-layer chromatography (TLC) analysis of the recovered surface-active extract obtained from *S. luridus* So3.2. The observed staining patterns indicated the presence of compounds compatible with lipid- and amino acid-related constituents in the extract.

TLC analysis also showed positive staining reactions consistent with lipid- and amino acid-associated components (Figure 10). Taken together, these assays provided preliminary chemical evidence of lipid- and peptide-associated components in the recovered extract.

**Figure 11:**
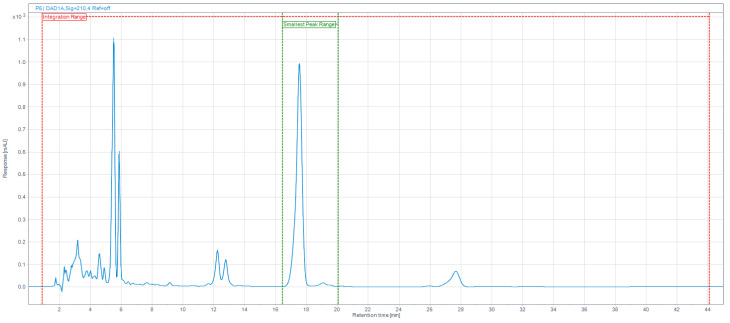
HPLC chromatogram of the crude biosurfactant extract obtained from *Streptomyces luridus* So3.2. The chromatographic profile showed multiple peaks, indicating that the recovered material consisted of several components. The fraction associated with peak 5 was selected for further concentration and downstream analytical evaluation.

HPLC analysis further indicated that the recovered material corresponded to a chemically heterogeneous extract rather than to a single purified compound. The chromatographic profile of the crude biosurfactant extract showed multiple resolved peaks, indicating the presence of several constituents in the sample (Figure 11). The fraction associated with peak 5 was selected after pre-concentration at 95 injections of the crude extract. After solvent removal, 7 mg of purified extract were obtained and dissolved in 5 mL of methanol, yielding a final concentration of 1.4 mg mL^−1^ (1400 mg L^−1^).

**Figure 12:**
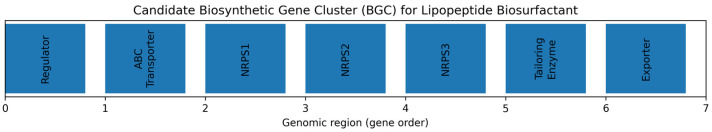
Schematic representation of the candidate biosynthetic gene cluster (BGC) associated with putative lipopeptide biosynthesis in *Streptomyces luridus* So3.2. The cluster includes genes encoding a regulator, ABC transporter, three NRPS modules, a tailoring enzyme, and an exporter, consistent with a non-ribosomal peptide biosynthetic system.

To strengthen the molecular interpretation, genome-mining analysis was performed for *S. luridus* So3.2. The assembled genome showed a total size of 8.67 Mb distributed in six contigs and a GC content of 70.7%, which is consistent with the genomic architecture typically observed in *Streptomyces* species. AntiSMASH analysis identified 38 biosynthetic gene clusters (BGCs), including multiple NRPS and NRPS-like regions (Figure 12). Among these, five regions were considered especially relevant to biosurfactant production, and region 10 (791,560–840,941 bp) was identified as the most plausible candidate for the biosynthesis of a lipopeptide-associated metabolite (Table 5). This region contains NRPS-associated genes compatible with the assembly of amphiphilic peptide-derived secondary metabolites.

**Figure 13:**
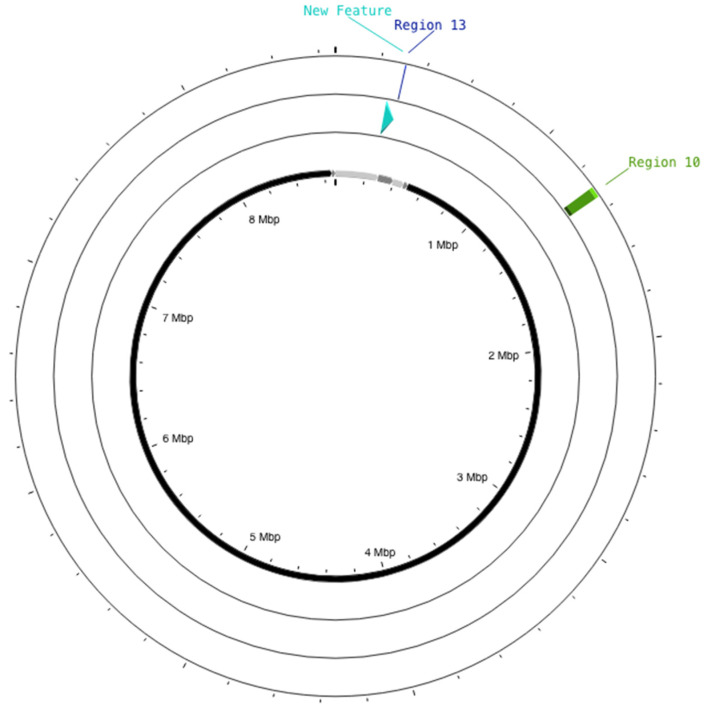
Circular genome map of *Streptomyces luridus* So3.2 showing the genomic positions of selected biosynthetic regions identified by antiSMASH. Candidate regions associated with biosurfactant-related biosynthesis, including Region 10 and Region 13, are highlighted on the genome map.

Based on the antiSMASH output, the candidate cluster is consistent with the biosynthesis of a lipopeptide-associated metabolite containing a hydrophobic fatty acid moiety linked to a short peptide backbone (Figure 13). Although this genomic evidence does not establish the final molecular structure of the recovered extract, it provides independent biosynthetic support for the production of a peptide-associated surface-active metabolite by *S. luridus* So3.2.

**Figure 14:**
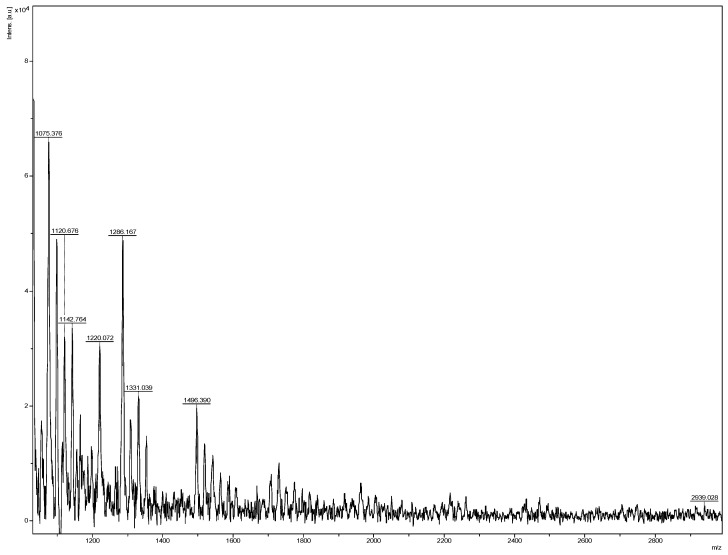
MALDI-TOF MS mass spectrum of the recovered surface-active extract produced by *Streptomyces luridus* So3.2. The spectrum showed a complex ion pattern with multiple peaks distributed mainly between *m*/*z* 900 and 1500.

MALDI-TOF MS analysis of the recovered surface-active extract revealed a complex ion profile, indicating that the sample was composed of multiple molecular species rather than a single purified compound (Figure 14). Major ion signals were detected at *m*/*z* 902.269, 910.604, 916.880, 933.670, 943.744, 987.260, 1027.744, 1032.113, 1075.376, 1098.477, 1120.676, 1142.764, 1220.072, 1286.167, 1308.105, 1331.039, 1496.390, and 1518.371, together with a minor high-mass signal at *m*/*z* 2939.028. Several peaks were distributed in closely spaced mass series, suggesting the presence of related molecular species within the extract.

## 3. Discussion

The present study demonstrates that *S. luridus* So3.2 can produce biosurfactants with relevant surface-active properties using recycled frying oils as carbon sources at 25 °C. This feature is particularly relevant for biotechnological applications, where there is increasing demand for bio-based surfactants produced through sustainable and economically feasible processes [5,25]. In contrast to many commonly reported biosurfactant-producing bacteria, such as *Pseudomonas* and *Bacillus*, which are often cultivated under more tightly controlled temperature conditions, the ability of *S. luridus* to maintain efficient biosurfactant production at 25 °C represents a potential advantage in terms of process simplification and reduced energy input. Previous evidence for *S. luridus* So3.2 was mainly based on bioemulsifying activity, which demonstrated the strain’s capacity to stabilize hydrophobic substrates [16]. In the present study, the observation of measurable surface tension reduction, together with the determination of CMC and CMD, supports the presence of biosurfactant-associated activity.

The use of recycled frying oils is also aligned with circular bioeconomy strategies aimed at valorizing agro-industrial residues while reducing dependence on refined feedstocks [26]. Recycled frying oils have been widely recognized as promising low-cost substrates for biosurfactant production because of their availability, reduced commercial value, and chemical complexity [27]. In this study, recycled frying oils not only supported growth but also enhanced biosurfactant-associated surface activity compared with commercial oils. Similar observations have been reported in other biosurfactant-producing systems, where waste-derived lipid matrices outperformed virgin oils in terms of either yield or functional activity, likely because thermal degradation products increase substrate accessibility and metabolic induction [27,28].

The experiments revealed that filtered and centrifuged recycled frying oil (CRO) consistently promoted higher emulsification indices, greater oil displacement, and stronger surface-active performance than the commercial oils evaluated. CRO also outperformed the merely filtered fraction (FRO), indicating that the centrifugation step influenced the functional response of *S. luridus* So3.2. This difference suggests that the degree of substrate refinement may play an important role in determining biosurfactant-associated performance [29]. Olive oil was predominantly enriched with monounsaturated fatty acids, whereas sunflower oil contained a higher proportion of polyunsaturated fatty acids. In contrast, the recycled frying oil exhibited an intermediate mono- and polyunsaturated lipid profile, together with the chemical modifications expected from repeated thermal processing [30,31]. Recycled frying oils generated through frying processes may contain oxidized fatty acids, free fatty acids, polymerized lipids, and other degradation products that could influence microbial metabolism and surface-active behavior [28,32]. This interpretation is consistent with the superior performance observed for CRO, suggesting that both the lipid composition of the recycled frying oil and the presence of thermally modified compounds contributed to the enhanced production of surface-active metabolites [28,33].

The absence of significant surface activity in uninoculated controls further confirms that the observed effects were attributable to microbial biosynthesis rather than to the intrinsic physicochemical properties of the oils alone. This distinction is important because it supports the interpretation that recycled frying oils are not merely passive carbon sources but active determinants of biosurfactant productivity and performance. In practical terms, this strengthens the value of recycled frying oils as functional substrates for microbial surfactant production rather than simply as low-cost feedstocks.

Kinetic monitoring showed that emulsification and oil displacement activities were detectable within the first 24 h of fermentation and increased progressively thereafter, coinciding with the accumulation of active biomass. The growth profile indicated that *S. luridus* So3.2 reached the stationary phase after approximately 72 h, while biosurfactant-associated activity continued to increase up to 96 h. These results suggest that biosurfactant synthesis is closely linked to primary metabolism rather than being strictly associated with late stationary phase or stress responses [34]. A comparable growth-associated pattern has been reported for *Bacillus amyloliquefaciens* C11 cultivated in a 2 L stirred-tank reactor, where biosurfactant production was detected at early stages of growth and accompanied rapid reductions in surface tension during active growth [35]. Likewise, *B. subtilis* RSL2 cultivated in a semicontinuous bioreactor using molasses as substrate produced 13.7 g L^−1^ biosurfactant under batch operation, also showing a close relationship between biomass development and surfactant accumulation [36]. Compared with those systems, the present study showed similarly early onset of surface activity but a lower estimated broth concentration (1.25 g L^−1^), which may reflect taxonomic differences, substrate composition, and the milder non-regulated cultivation conditions employed here.

Growth-associated biosurfactant production is advantageous for industrial processes, since it simplifies fermentation control and may shorten production cycles. This behavior is also consistent with earlier observations for *S. luridus* So3.2, which was previously described as a producer of compounds with strong bioemulsifying potential when cultivated on hydrocarbon-rich substrates [16]. In that context, the present study not only confirms the metabolic robustness of this strain but also expands its relevance by demonstrating that a recycled frying oil substrate can sustain both microbial growth and progressive accumulation of surface-active metabolites under process-oriented conditions.

The biosurfactant produced under optimized conditions exhibited effective surface tension reduction and high emulsification capacity at relatively low concentrations, as reflected by the determined CMC and CMD values. Low CMC values indicate that a surfactant can reduce surface/interfacial tension and form micelles at relatively low bulk concentrations, so smaller amounts are required to achieve effective emulsification and solubilization [37]. This behavior is generally associated with strong surface activity in microbial biosurfactants and good emulsifying indices (e.g., E24% > 50–70%), including rhamnolipids, surfactin-like lipopeptides, and other amphiphilic microbial metabolites [38].

The surface activity observed in the present study is competitive when compared with recent biosurfactant systems produced from low-cost substrates. The CMC obtained (29 mg L^−1^) was lower than the 51.5 mg L^−1^ reported for a lipopeptide biosurfactant produced by *B. amyloliquefaciens* C11, indicating that the biosurfactant produced by *S. luridus* So3.2 reached efficient interfacial activity at a lower concentration [35]. However, the minimum surface tension achieved in the present study under reactor conditions (35.5 mN m^−1^) was less pronounced than the 28 mN m^−1^ reported for the *B. amyloliquefaciens* system, suggesting that while the biosurfactant from *S. luridus* is highly efficient in terms of micelle formation threshold, its absolute surface tension reduction remains moderate relative to some *Bacillus*-derived lipopeptides. A similar contrast is observed when compared with the glycolipid produced by *Candida mogii*, which reduced surface tension to 28.66 mN m^−1^ but showed a much higher CMC of 0.8 g L^−1^ under optimized conditions [39]. Therefore, the biosurfactant produced by *S. luridus* So3.2 appears to combine relatively high concentration efficiency with moderate terminal surface-tension reduction, a profile that may still be highly attractive for applications in which low dosage and strong emulsifying capacity are prioritized.

Reported CMC values for lipopeptide and lipid-associated biosurfactants typically range from tens to several hundreds of milligrams per liter, depending on molecular structure and purity [40]. The CMC obtained in the present study falls within the lower end of this range, supporting the interpretation that the biosurfactant is a high-efficiency surface-active compound. Moreover, the significant positive correlation observed between emulsification and oil displacement activities indicates functional coherence between these surface activity parameters, reinforcing the usefulness of both variables as complementary indicators of biosurfactant-mediated activity [41]. As mentioned by Sari et al. [42], efficient biosurfactants typically display low CMC values together with substantial reductions in water surface tension, often to approximately ~34–40 mN m^−1^ or lower, which is consistent with the overall performance observed. 

The application of response surface methodology enabled the identification of cultivation conditions that maximized emulsification activity while maintaining process robustness. The statistical significance of the model, together with the high coefficient of determination and the non-significant lack-of-fit, confirms the suitability of RSM for optimizing biosurfactant production using recycled frying oils as complex substrates.

Among the evaluated variables, inoculum and carbon source concentrations exerted the most significant influence on emulsification performance, highlighting the importance of biomass availability and substrate accessibility in biosurfactant biosynthesis [43]. Similar trends have been reported in other waste-based biosurfactant systems optimized using response surface methodology, in which substrate concentration was identified as a key determinant of surface activity and process efficiency [44]. A useful recent comparison is provided by *C. mogii* cultivated in licuri oil, where Central Composite Design yielded a predictive model with R^2^ = 0.9451 and surface tension values reduced to 28.66 mN m^−1^ under optimized conditions [33]. In the present study, the quadratic model showed an even higher explanatory capacity (R^2^ = 0.9828), but the most responsive variable was E24 rather than absolute surface tension. This difference suggests that, in the *S. luridus* So3.2 system, optimization was more strongly reflected in emulsion-forming capacity than in maximal interfacial tension collapse. In such systems, the use of complex, low-cost substrates requires careful balancing between microbial growth and metabolic induction to achieve stable and reproducible surface-active responses. The reproducibility observed at the central points in the present study further supports the robustness of the optimized process and reduces uncertainty associated with subsequent reactor-based validation.

The stirred-tank reactor experiments represent a critical step toward technological relevance, since they address oxygen availability and mixing effects that cannot be adequately captured in shake-flask systems. The results indicate that moderate aeration combined with intermediate agitation produced the most favorable balance of emulsification, oil displacement, and surface tension reduction. In contrast, non-aerated conditions consistently resulted in lower surface-active performance, in agreement with the importance of oxygen transfer and hydrodynamic conditions for aerobic metabolite production [45,46].

The reactor-scale results are also quantitatively favorable when compared with other recent bioreactor-based biosurfactant studies, although the optimal engineering windows differ across systems. In the present work, the best 2 L stirred-tank condition yielded E24 values of 83.3%, oil displacement halos up to 12.50 cm, and surface tension of 35.5 mN m^−1^ at 0.5 vvm and 150 rpm. By contrast, an air-lift bioreactor process with *B. atrophaeus* identified 1.00 vvm and 34 °C as the best operational condition, but the resulting emulsification index was lower at 66.9% [47]. These differences suggest that the *S. luridus* system achieved stronger emulsification performance under comparatively moderate aeration, which may be advantageous from an operational perspective. At the same time, *Bacillus*-based systems may still achieve stronger absolute surface tension reductions, indicating that different microbial platforms may be optimized toward distinct functional outputs.

These observations are consistent with the known sensitivity of *Streptomyces* physiology to oxygen availability and hydrodynamic conditions. The results obtained under different aeration and agitation regimes indicate that oxygen supply and mixing efficiency are central to biosurfactant-associated surface activity. Moderate aeration combined with intermediate agitation resulted in improved emulsification, oil displacement, and reduced surface tension, whereas non-aerated conditions consistently showed lower performance [48]. These findings support the feasibility of controlling biosurfactant production in stirred-tank systems through operational parameters that directly affect oxygen availability, without requiring extreme aeration or agitation conditions. Importantly, the maintenance of strong surface activity during cultivation in the 20 L reactor further demonstrates that the process retains functional performance beyond flask-level experiments, supporting the technical feasibility of biosurfactant production under increased reactor volume.

From a molecular perspective, the functional behavior observed in the present study is consistent with a lipopeptide-associated biosurfactant. Lipopeptide biosurfactants combine strong surface activity with considerable structural diversity, and robust assignment commonly relies on complementary analytical workflows rather than on a single method. FTIR and TLC analyses provided preliminary chemical evidence of lipid- and peptide-associated components in the recovered extract. The FTIR profile obtained in the present study is also broadly consistent with the pattern previously reported for *S. luridus* So3.2 by Lamilla et al. [16], who described absorption bands associated with fatty acid-related moieties, whereas HPLC revealed multiple chromatographic peaks and MALDI-TOF MS showed a heterogeneous mass profile, together supporting the interpretation of a chemically complex surface-active extract [35,49,50,51,52]. Together, these interpretations are consistent with recent reports in which chromatographic and mass-spectrometric approaches were used to support lipopeptide identification and to distinguish between isoforms or homologous species [35].

In addition, the genome-mining results further strengthen this interpretation at the biosynthetic level. The detection of multiple NRPS and NRPS-like regions, including a candidate cluster potentially associated with peptide-derived amphiphilic metabolites, is compatible with the production of lipopeptide-associated compounds, although genomic evidence alone does not resolve final molecular structure. A similar integrative framework has been recently applied in waste-oil-based biosurfactant studies, where bioprocess performance, molecular profiling, and biosynthetic evidence were interpreted together while still recognizing the need for deeper structural confirmation [53]. Therefore, the most appropriate interpretation of the present dataset is that *S. luridus* So3.2 produces a putative lipopeptide-associated surface-active extract.

From a process perspective, the present system occupies an intermediate but relevant position among recent biosurfactant production platforms. It does not yet reach the very low surface tension values reported for some *Bacillus* or yeast-based systems, such as 28.0–28.7 mN m^−1^, nor the volumetric production of 13.7 g L^−1^ achieved in a semicontinuous *B. subtilis* bioreactor [35,36,39]. Nevertheless, it combines several desirable traits within a single platform: the use of recycled frying oil as substrate, detectable activity within 24 h, E24 values above 80%, a low CMC of 29 mg L^−1^, and validation in stirred-tank reactors up to 20 L. This combination is comparatively uncommon in the recent literature, where substrate valorization, statistical process design, and reactor validation are often reported separately rather than within the same study.

The combination of effective surface activity, low CMC, production under mild conditions, and the use of recycled substrates positions the biosurfactant produced by *S. luridus* So3.2 as a promising candidate for further formulation studies. Recent biosurfactant research emphasizes the need to bridge fermentation performance with downstream validation, including emulsion stability, compatibility with formulation matrices, and safety assessments [49]. While such evaluations were beyond the scope of the present work, the results reported here provide a solid bioprocess foundation for subsequent application-driven studies. Future work should therefore focus on formulation testing and toxicological assays to fully assess the suitability of this biosurfactant for biotechnological applications.

## 4. Materials and Methods

### 4.1. Microorganism and Inoculum Preparation

*Streptomyces luridus* So3.2, isolated from Antarctic rhizospheric soil and taxonomically characterized by Lamilla et al. [16], was obtained from the culture collection of the Environmental Biotechnology Laboratory II, Universidad de La Frontera, Chile. The strain was reactivated from cryopreserved stocks and streaked onto M1 (2.0 g L^−1^ peptone; 4.0 g L^−1^ yeast extract; 10.0 g L^−1^ starch; 18.0 g L^−1^ agar; pH 7.0 ± 0.2) and LB (10.0 g L^−1^ peptone; 5.0 g L^−1^ yeast extract; 5.0 g L^−1^ sodium chloride; pH 7.0 ± 0.2) agar plates. Plates were incubated at 25 °C for 7 days until sporulated colonies were obtained, and colony morphology was verified by optical microscopy.

For inoculum preparation, a single isolated colony was transferred into 250 mL Erlenmeyer flasks containing 100 mL of LB broth and incubated at 25 °C at 130 rpm for 96 h. This culture was used as inoculum for subsequent biosurfactant production experiments and subsequent assays.

### 4.2. Carbon Sources and Oil Pre-Treatment

Different vegetable oils of commercial and recycled origin were evaluated as potential low-cost carbon sources for biosurfactant production by *S. luridus* So3.2 (Table 6). Commercial oils included sunflower oil (SFO) (Chef 100% sunflower oil) and olive oil (OO) (Calendula), whereas recycled frying oil was obtained from a local fast-food restaurant. According to product label information, olive oil contained predominantly monounsaturated fatty acids (73.0 g/100 mL), with lower amounts of saturated (11.0 g/100 mL) and polyunsaturated fatty acids (8.0 g/100 mL). Sunflower oil contained 10.0 g/100 mL saturated, 28.0 g/100 mL monounsaturated, and 54.0 g/100 mL polyunsaturated fatty acids. Recycled frying oil contained 8.1 g/100 mL saturated, 46.7 g/100 mL monounsaturated, and 37.2 g/100 mL polyunsaturated fatty acids, with linoleic acid representing 66% of total fatty acids. The measured pH values were 5.5 for OO, 6.5 for SFO, and 4.5 for recycled frying oil (Table 1).

The recycled frying oil was processed using two sequential treatments. First, a filtration step was performed by passing 500 mL of recycled frying oil through multilayer sterile gauze to remove suspended solids and food residues. This fraction was designated as filtered recycled frying oil (FRO). Subsequently, a more refined fraction was obtained by centrifuging 100 mL of the FRO fraction in 50 mL Falcon tubes at 10,000 rpm for 10 min. The recovered supernatant was designated as filtered and centrifuged recycled frying oil (CRO).

All oil fractions were stored at room temperature in sterile containers until use as carbon sources in subsequent biosurfactant production and selection experiments.

### 4.3. Flask-Scale Biosurfactant Production and Kinetic Monitoring

Biosurfactant production by *S. luridus* So3.2 was evaluated using different vegetable oils as carbon sources, including recycled frying oils and commercial oils. The recycled frying oil fractions consisted of FRO and CRO, while commercial oils included SFO and OO.

Experiments were conducted in 250 mL Erlenmeyer flasks containing 100 mL of minimal medium (MM (6.0 g L^−1^ Na_2_HPO_4_; 3.0 g L^−1^ KH_2_PO_4_; 0.5 g L^−1^ NaCl; 1.0 g L^−1^ NH_4_Cl; 0.24 g L^−1^ MgSO_4_·7H_2_O)) supplemented with the selected oil at a concentration of 1.5% (*w*/*v*) as the sole carbon source. Culture media were sterilized by autoclaving at 121 °C for 15 min, cooled to room temperature, and inoculated with 3% (*w*/*v*) of the bacterial inoculum prepared as described previously.

Cultures were incubated at 25 °C under orbital shaking at 150 rpm for 96 h. At the end of the fermentation period, cultures were centrifuged at 10,000 rpm for 10 min to obtain cell-free supernatants.

Biosurfactant production was initially assessed by determining emulsification index after 24 h (E24%) and water surface tension reduction in the cell-free supernatant as primary selection parameters. Carbon sources yielding surface tension values lower than 38 mN m^−1^ and emulsification indices higher than 70% were selected for subsequent bioreactor experiments.

Kinetic monitoring of biosurfactant production was conducted following the methodology described by Lamilla et al. [16]. Cultures were cultivated in 250 mL Erlenmeyer flasks containing 100 mL of minimal medium supplemented with filtered and centrifuged recycled frying oil (CRO) at 1.5% (*w*/*v*) and inoculated with 3% (*w*/*v*) bacterial inoculum. Incubation was carried out at 25 °C under orbital shaking at 150 rpm for 96 h. Samples were collected at 18 h, 24 h, and subsequently every 24 h until the end of cultivation to evaluate emulsification activity (E24%), oil displacement activity, and biomass concentration. Biomass concentration (g L^−1^) was estimated using the following calibration equation:

$$\mathrm{Biomass}\;\mathrm{concentration}=\frac{{\mathrm{OD}}_{600}-0.0251}{1.8292}$$

### 4.4. Experimental Design by Central Composite Design (CCD-RSM)

The Central Composite Design (CCD) based on response surface methodology (RSM) was applied to evaluate the effects and interactions of key cultivation variables on biosurfactant production by *S. luridus* So3.2 using the selected low-cost carbon source.

Three independent variables were evaluated: pH (range 6–8), inoculum concentration (1–3% *w*/*v*), and carbon source concentration (1–2% *w*/*v*). Experiments were conducted at ambient temperature (25 °C) under orbital agitation at 160 rpm in 250 mL Erlenmeyer flasks containing 100 mL of minimal medium (MM). Fermentations were carried out for 96 h.

A total of 17 experimental runs, including central-point replicates, were performed according to the CCD matrix. For the RSM analysis, the emulsification index (E24%) was used as the response variable for optimization, whereas oil displacement activity was evaluated only as a complementary surface-activity parameter.

### 4.5. Surface Activity Assays

The surface activity of the biosurfactant produced by *S. luridus* So3.2 was evaluated using cell-free supernatants obtained after centrifugation of the fermentation broths at 10,000 rpm for 10 min. All assays were performed in triplicate.

Oil displacement activity was assessed as a method for biosurfactant surface activity following the procedure described by Morikawa et al. [54]. Briefly, 40 mL of distilled water was added to a Petri dish (15 cm diameter), and 15 µL of vegetable oil was deposited onto the water surface to form a thin oil layer. Subsequently, 15 µL of cell-free supernatant was carefully added to the oil layer, and the diameter of the clear zone formed was measured in centimeters. Distilled water was used as a negative control, while Tween 80 (1% *v*/*v*) served as the positive control. The comparison with Tween 80 should be interpreted only as a functional benchmark for surface-active performance, not as evidence of chemical or structural equivalence with the recovered microbial extract. In addition, culture medium without biosurfactant and culture broth obtained using olive oil in the same proportion were included as controls.

Emulsification activity was determined according to the method described by Sriram et al. [55], with minor modifications. Equal volumes (2 mL) of cell-free supernatant and vegetable oil were transferred into glass test tubes and vortexed vigorously for 4 min. The mixtures were then allowed to stand at room temperature for 24 h. The emulsification index (E24%) was calculated as the ratio between the height of the emulsion layer and the total height of the mixture, multiplied by 100. All assays were conducted in triplicate.

Surface tension reduction was evaluated using cell-free supernatants obtained after centrifugation, employing a digital tensiometer (HZL-3, Huazheng, China) according to the method described by George and Jayachandran [56]. Measurements were performed at room temperature (25 ± 1 °C) using 20 mL of cell-free supernatant, and distilled water (72 mN m^−1^) was used as a negative control and for instrument calibration. All measurements were performed in triplicate.

The critical micelle concentration (CMC) of the crude biosurfactant was determined at 25 °C using serial dilutions prepared from biosurfactant solutions with concentrations ranging from 0 to 1000 mg L^−1^. Surface tension was measured at each dilution after calibration of the tensiometer with distilled water. The CMC was defined as the concentration corresponding to the breakpoint in the surface tension versus biosurfactant concentration curve, following the approach described by Safari et al. [57].

Additionally, the Critical Micelle Dilution (CMD) was determined by identifying the dilution factor at which the minimum stable surface tension was achieved. The CMD value was used to estimate the biosurfactant concentration in the fermentation broth by multiplying the CMC value by the corresponding dilution factor.

### 4.6. Stirred-Tank Bioreactor Experiments

Biosurfactant production by *S. luridus* So3.2 was further evaluated under stirred-tank reactor conditions to assess process performance and scalability. Fermentation assays were conducted in glass stirred-tank reactors with total capacities of 2 L and 20 L, operating under batch mode.

Laboratory-scale experiments were initially performed in a 2 L glass stirred-tank reactor with a working volume of 1 L. The reactor vessel and all accessories were sterilized by autoclaving at 121 °C for 15 min prior to inoculation. Cultivations were carried out using minimal medium supplemented with the selected carbon source under the optimized conditions identified in flask-level experiments. Aeration and agitation were evaluated as operational variables, applying aeration rates of 0, 0.5, and 0.9 vvm and agitation speeds of 100, 150, and 200 rpm. Fermentations were conducted at 25 °C for 96 h. At the end of the cultivation period, samples were collected and centrifuged at 10,000 rpm for 10 min to obtain cell-free supernatants, which were subsequently analyzed for emulsification index (E24%), oil displacement activity, and surface tension reduction according to the methods described in the corresponding analytical Section 4.5.

Biosurfactant production was subsequently evaluated in a 20 L stirred-tank reactor with a working volume of 15 L. The reactor was sterilized prior to use following standard laboratory procedures. Fermentations were carried out under the same optimized cultivation conditions identified at the flask scale and applied in the 2 L reactor. Cultivation was performed at 25 °C for 72 h under batch mode. Surface activity parameters, including emulsification index, oil displacement activity, and surface tension reduction, were monitored during fermentation. At the end of cultivation, the broth was processed to obtain cell-free supernatants, and the recovered biosurfactant was lyophilized for further analysis.

### 4.7. Chemical, Analytical, and Genomic Characterization of the Surface-Active Extract

The extract selected for chemical characterization was obtained from the cell-free supernatant of cultures grown with filtered and centrifuged recycled frying oil (CRO), which was selected based on its superior surface-activity performance. The extract was recovered by solvent extraction using chloroform:methanol (2:1, *v*/*v*). The organic phase was collected and evaporated under reduced pressure, and the dried extract was stored at room temperature until analysis.

Fourier Transform Infrared (FTIR) spectroscopy was performed to identify the main functional groups present in the recovered extract. Spectra were recorded using an FTIR spectrometer equipped with an ATR accessory (Agilent Cary 630, Agilent Technologies, Santa Clara, CA, USA) in the range of 4000–500 cm^−1^ and at 4 cm^−1^ resolution. The spectra were analyzed to identify characteristic absorption bands associated with lipid- and peptide-related moieties.

Thin-layer chromatography (TLC) was conducted to evaluate the chemical nature of the recovered extract. Samples were spotted onto silica gel 60 F254 TLC plates and developed using chloroform:methanol as the mobile phase. After development, the plates were visualized using reagents selective for lipid- and amino acid-associated components. Ninhydrin was used to detect amino acid-containing compounds, whereas iodine vapor was used for lipid visualization.

High-performance liquid chromatography (HPLC) analysis was performed using an analytical Merck Hitachi LC system equipped with a diode array detector (DAD). Chromatographic separation was carried out on a Purospher^®^ STAR RP-18 endcapped column (5 µm, Hibar^®^ RT 250–4.6 mm). The mobile phase consisted of acetonitrile (phase A) and water containing 3.8 mM trifluoroacetic acid (phase B). The chromatographic run was performed under isocratic conditions (80% A/20% B) at a flow rate of 1 mL min^−1^, a column temperature of 30 °C, an injection volume of 20 µL, and a total run time of 45 min. The fraction corresponding to peak 5 was selected after repeated chromatographic injections of the crude extract for further concentration and downstream analytical evaluation.

The molecular weight and congener profile of the crude biosurfactant extract were determined by matrix-assisted laser desorption/ionization time-of-flight mass spectrometry (MALDI-TOF MS), following analytical approaches commonly used for biosurfactant characterization and congener profiling [58,59]. Briefly, 1 µL of the extract was spotted onto a stainless-steel MALDI target plate (Bruker Daltonics, Bremen, Germany) and mixed with 1 µL of a saturated solution of α-cyano-4-hydroxycinnamic acid (CHCA) prepared in 50% acetonitrile containing 2.5% trifluoroacetic acid. Mass spectra were acquired using a MALDI-TOF MS Autoflex Speed instrument (Bruker Daltonics, Bremen, Germany) equipped with a Smartbeam laser source (334 nm), following the methodology described by Lamilla et al. [60]. Analyses were performed in positive-ion linear mode using an acceleration voltage of 20 kV and an extraction delay of 220 ns. Each spectrum represented an average of 1200 laser shots. Spectra were acquired over the range of 500–3000 *m*/*z*. Prior to sample analysis, the instrument was externally calibrated using the ProteoMass Peptide and Protein MALDI-MS Calibration Kit (Sigma-Aldrich). Spectral acquisition and data processing were performed using FlexControl 1.4 software (Bruker Daltonics, Bremen, Germany).

Genome-mining analysis was performed to identify biosynthetic gene clusters potentially associated with biosurfactant production by *Streptomyces luridus* So3.2. Genomic sequences were analyzed using antiSMASH 7.0 [61], and candidate biosynthetic regions were screened based on the presence of NRPS and NRPS-like domains. Regions considered potentially associated with biosurfactant-related biosynthesis were selected for further interpretation.

### 4.8. Statistical Analysis

All experiments were conducted at least in triplicate, and results are expressed as mean values ± standard deviation. Statistical analyses were performed using JMP^®^, Version 17 (SAS Institute Inc., Cary, NC, USA). For response surface methodology (RSM) experiments, analysis of variance (ANOVA) was applied to evaluate the significance of fitted models and individual factors. Model adequacy was assessed based on the coefficient of determination (R^2^), adjusted R^2^, lack-of-fit testing, and *p*-values. Differences were considered statistically significant at *p* < 0.05.

When significant differences among treatments were detected, mean comparisons were performed using Tukey’s HSD post hoc test. Differences were considered statistically significant at *p* < 0.05. Correlation analysis between surface activity parameters, including emulsification index (E24%) and oil displacement activity, was performed to assess the relationship between response variables.

## 5. Conclusions

This study demonstrated that *Streptomyces luridus* So3.2 can produce a surface-active extract using recycled frying oil as a low-cost carbon source under mild cultivation conditions. Among the evaluated substrates, filtered and centrifuged recycled frying oil (CRO) was selected as the most suitable waste-derived substrate based on its combined performance in emulsification, oil displacement, and relevant surface activity.

Response surface methodology identified cultivation conditions that improved emulsification performance at the flask scale, and these optimized conditions were subsequently applied in stirred-tank reactor assays. The 2 L reactor experiments showed that selected aeration/agitation regimes influenced surface-active performance, while the 20 L assay indicated that these functional properties could be maintained under increased working volume.

Chemical and molecular analyses provided preliminary support for the nature of the recovered product. FTIR and TLC indicated lipid- and peptide-associated components, while HPLC and MALDI-TOF MS revealed that the recovered material corresponds to a chemically heterogeneous extract rather than a single purified compound. In addition, genome mining identified multiple NRPS and NRPS-like biosynthetic regions, including a candidate cluster potentially associated with peptide-derived amphiphilic metabolites.

Overall, these results support the interpretation that *S. luridus* So3.2 produces a putative lipopeptide-associated surface-active extract from recycled frying oil. Although further structural confirmation is still required to fully define the molecular identity of the recovered material, the present study demonstrates the potential of this strain and substrate combination for biosurfactant production and functional performance at a reactor scale. 

In process.

## Figures and Tables

**Table 1:** Surface tension (mN m^−1^) and final pH measured in the cell-free supernatants obtained from different carbon sources and controls.

Treatment	Surface Tension (mN m^−1^)	Final pH
Olive oil (OO)	33.5 ± 0.2 ^d^	6.2
Sunflower oil (SFO)	38.3 ± 0.6 ^c^	6.9
Filtered recycled frying oil (FRO)	37.6 ± 0.3 ^c^	7.0
Filtered and centrifuged recycled frying oil (CRO)	37.6 ± 0.2 ^c^	6.9
Distilled water control	70.1 ± 0.0 ^a^	5.7
Culture medium with oil	58.5 ± 0.0 ^b^	6.9

Different letters indicate significant differences according to one-way ANOVA followed by Tukey’s HSD test (*p* < 0.05).

**Table 2:** Central Composite Design (CCD) matrix of independent variables and their corresponding experimental and predicted yields of the emulsification index (E24%).

Run	Factors			Response Emulsification (E24%)	
	A	B	C	Exp. Value	Pred. Value
1	8	3	1	59	60.6
2	7	2	2	69	70.8
3	7	3	1.5	60	60.0
4	6	1	2	60	58.0
5	8	2	1.5	65	63.6
6	8	1	2	65	65.7
7	7	2	1.5	60	61.3
8	7	2	1	69	68.2
9	8	1	1	61	60.7
10	7	1	1.5	52	53.0
11	8	3	2	82	81.3
12	6	1	1	73	73.5
13	6	3	1	73	72.0
14	7	2	1.5	61	61.3
15	7	2	1.5	65	61.3
16	6	3	2	72	72.1
17	6	2	1.5	63	65.4

**Table 3:** Analysis of variance (ANOVA) for the quadratic model.

Source	Degrees of Freedom	Sum of Squares	Mean Square	F-Ratio
Model	9	305.61051	33.9567	15.3960
Error	7	15.43890	2.2056	
Total	16	321.04941		
			Prob > F	0.0008 *

* Indicates that the quadratic model was statistically significant at *p* < 0.05.

**Table 4:** Surface activity of *S. luridus* So3.2 in different aeration and agitation conditions in a 2 L stirred-tank reactor.

Aeration (vvm)	Agitation (rpm)	%E24	Oil Displacement (cm)	Surface Tension mN m^−1^
0	100	79.4	7.9 ± 0.56	38.5
0.5	150	83.3	12.50 ± 0.86	35.5
0	150	82	8.76 ± 0.15	38.5
0.9	200	83.3	10.49 ± 1.02	37.2

**Table 5:** antiSMASH regions identified in the genome of *Streptomyces luridus* So3.2 and considered potentially associated with biosurfactant biosynthesis.

antiSMASH Region	Position (bp)	Type
Region 3	270,041–322,512	NRPS
Region 10	791,560–840,941	NRPS
Region 13	1,366,712–1,413,684	NRPS
Region 15	1,627,309–1,671,311	NRPS-like
Region 27	5,415,895–5,459,729	NRPS-like

**Table 6:** Physicochemical characteristics of vegetable oils used as carbon sources.

Oil Source	Saturated Fat (g/100 mL)	Monounsaturated Fat (g/100 mL)	Polyunsaturated Fat (g/100 mL)	Main Fatty Acid	pH
Olive oil (OO)	11.0	73.0	8.0	Oleic acid	5.5
Sunflower oil (SFO)	10.0	28.0	54.0	Linoleic acid	6.5
Recycled frying oil (FRO-CRO)	8.1	46.7	37.2	Linoleic acid	4.5

## Data Availability

Data are contained within the article.
